# Influence of intermediate abutment height and timing of placement on marginal bone loss in single implant-supported crowns: a 12-month follow-up randomized clinical trial

**DOI:** 10.1007/s00784-025-06364-8

**Published:** 2025-05-08

**Authors:** Jacobo Quintas-Hijós, Esteban Pérez-Pevida

**Affiliations:** 1 Department of Surgery, Faculty of Medicine, University of Salamanca, Salamanca, Spain; 2 Department of Surgery, Faculty of Sports and Health Sciences, University of Zaragoza, Huesca, Spain; 3 DENS-ia Research Group, Department of Health Sciences, Miguel de Cervantes European University, Valladolid, Spain

**Keywords:** Dental implants, Single tooth dental implant, Dental implant-abutment design, Implant-supported, Bone resorption

## Abstract

**Objectives:**

To determine the most effective combination of abutment height and timing of placement in reducing marginal bone loss (MBL).

**Materials and methods:**

54 patients received at least one single screw-retained crown on an implant replacing a posterior tooth (60 implants). Implants were divided into six groups based on intermediate abutment height (1.5 mm, 2 mm, 3 mm) and timing of placement (immediate: surgery 1; delayed: surgery 2): Group **A3I** (height 3, surgery 1), Group **A2I** (height 2, surgery 1), Group **A15I** (height 1.5, surgery 1), Group **A3D** (height 3, surgery 2), Group **A2D** (height 2, surgery 2), Group **A15D** (height 1.5, surgery 2). Mesial and distal linear radiographic measurements were taken at five follow-up points: implant surgery, crown placement (T1), and 3 (T2), 6 (T3), and 12 months after loading (T4). Partial and total MBL were compared between groups.

**Results:**

After 12 months, the lowest MBL was found in groups **A3I** (0.13 ± 0.11 mm) and **A2I** (0.24 ± 0.11 mm), with no statistical difference between them. Groups **A15I** (0.70 ± 0.12 mm), **A3D** (0.66 ± 0.11 mm), **A2D** (0.62 ± 0.12 mm), and **A15D** (0.78 ± 0.11 mm) showed significantly higher MBL than groups 1 and 2, with no statistical difference among them.

**Conclusions:**

Immediate abutments of 2–3 mm resulted in lower MBL compared to 1.5 mm immediate abutments or any delayed abutments.

**Clinical Relevance:**

This study provides data on the optimal combination of intermediate abutment height and placement timing in preventing MBL.

**Clinical Trial Registration:**

Clinical trial registration number: NCT06667531. link: https://clinicaltrials.gov/study/NCT06667531.

## Introduction

Early marginal bone loss (MBL) is a variable, non-infectious remodeling process that occurs within the first year following implant placement. Its etiology is multifactorial, influenced by both surgical and prosthetic factors [1]. Longitudinal analysis of MBL rates is more predictive, clinically relevant, and informative than cross-sectional analysis. With regard to peri-implant MBL, initial rates indicate the likelihood of reaching higher values in the future, regardless of the etiology of crestal bone los [2].

Factors influencing MBL can be classified into local, systemic, and **life-style** factors [3]. Local implant-related factors include macroscopic design features such as platform switching [4], microscopic design characteristics such as surface roughness [5], implant diameter [6], connection type [7], surgical trauma [8], and apico-coronal position [9]. Local patient-related factors included bone quantity [10], bone density [11], and soft tissue thickness [12]. Systemic factors encompass conditions such as diabetes [13], previous periodontal disease [14], IL-1 polymorphism [15], Life-style factors include plaque control [16] and smoking [17].

The intermediate abutment is a transmucosal component that connects dental implants to prostheses, facilitating the transmission of masticatory forces. Simultaneously, it serves as the key component that protects implants from the contaminated oral environment [18]. The internal conical connection provides superior hermetic sealing and stability at the implant-abutment interface, making it preferable for preserving peri-implant crestal bone levels [19].

Like natural teeth, there is a supracrestal tissue attachment around implants, providing a biological seal against bacterial pathogens and the invasion of food debris at the implant-tissue interface. The height of the intermediate abutment could influence MBL by interacting with the space required to restore biological width [20]. Greater MBL may be observed for prosthetic abutments less than 2 mm in height compared to those greater than 2 mm, following a non-linear trend, with higher MBL occurring in the first 6 months post-loading compared to the subsequent 12 months [21].

Additionally, repeated connection and disconnection of healing/provisional abutments after implant placement can compromise the mucosal barrier and induce apical migration of the connective tissue attachment, accompanied by underlying bone remodeling. Abutment manipulation can thus lead to mechanical injury to the soft tissue barrier, causing it to re-establish at a more apical position, resulting in marginal bone resorption [22]. The use of definitive abutments at the time of implant placement has been proposed as a strategy to enhance peri-implant tissue stability. This approach aims to minimize disruption at the implant-abutment interface, which may help preserve the marginal bone levels over time [23]. By maintaining a stable implant-abutment connection, the initial hard tissue healing process could be favorably influenced, potentially contributing to long-term peri-implant health [24].

No clinical studies in the literature have determined the combined influence of intermediate abutment height and placement timing on MBL for single screw-retained crowns. This randomized clinical trial aims to evaluate changes in peri-implant marginal bone levels during the first year of function around platform-switched implants placed 1.5 mm subcrestally, connected to intermediate abutments of heights 1.5, 2, and 3 mm, which are placed either immediately at the time of the first surgery or delayed until the second surgery. The primary objective was to analyze which combination of abutment height and placement timing is most effective in reducing peri-implant MBL after 12 months of loading, while also examining the most effective combination over time. **The study hypothesis was that the immediate placement (first-stage surgery) of a long abutment (≥ 2 mm) would result in less marginal bone loss compared to the delayed placement (second-stage surgery) of a short abutment (< 2 mm).**

## Materials and methods

### Study design

This is a six-arm, parallel, randomized clinical trial. The study protocol was reviewed and approved by the Research Ethics Committee of the Autonomous Community of Aragon (CEICA, C.I. PI22/324). All participants provided written informed consent in accordance with the 1975 Helsinki Declaration prior to the commencement of treatment. The study was registered at www.clinicaltrials.gov under the identifier NCT06667531. The results of this randomized clinical trial are reported in compliance with ID-COSM [25] and adhere to the CONSORT checklist.

### Trial population

**Fig. 1:**
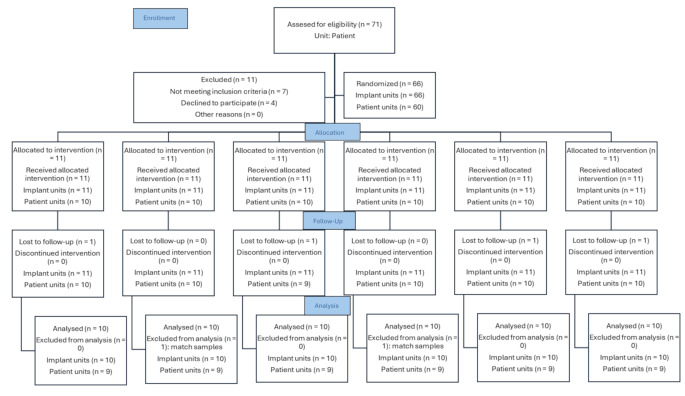
CONSORT guidelines flowchart

All participants were selected and followed up at the same private center (Quintas Hijós Clínica Dental) between June 30, 2022, and June 28, 2024. The selection was based on the following inclusion criteria: (a) healthy adults, (b) Plaque Index (PI) < 10%, (c) Bleeding on Probing (BOP) < 10%, (d) absence of chronic periodontal diseases (periodontitis) or acute periodontal conditions (periodontal abscess), (e) presence of a fully healed socket with no bone defects, (f) sufficient bone height (10 mm) and width (1.5 mm), (g) need for restoration of a tooth in the posterior maxillary or mandibular region (premolars and molars), and (h) enrollment in a periodontal maintenance program. The exclusion criteria included: (a) history of systemic disease or radiotherapy contraindicating bone surgery, (b) pregnant or breastfeeding women, (c) smokers of more than 10 cigarettes per day, (d) parafunctional habits, (e) alcohol or drug abuse, (f) narrow interproximal spaces (less than 9 mm), (g) placement of implants flapless or post-extraction, and (h) insertion torque during implant placement below 35 Ncm. A total of 71 subjects were initially selected. Seven were excluded for not meeting the inclusion criteria, and an additional four declined to participate in the study. Sixty-six implants placed in 60 subjects were randomly allocated into one of six treatment groups based on the height (1.5 mm, 2 mm, 3 mm) and timing of placement (immediate: time 1; delayed: time 2) of the intermediate abutment: group **A3I** (height 3 mm; time 1; 11 implants), group **A2I** (height 2 mm; time 1; 11 implants), group **A15I** (height 1.5 mm; time 1; 11 implants), group **A3D** (height 3 mm; time 2; 11 implants), group **A2D** (height 2 mm; time 2; 11 implants), group **A15D** (height 1.5 mm; time 2; 11 implants). Four subjects were lost to follow-up, and two additional participants were excluded through simple randomization to ensure equal sample sizes across the six groups. Ultimately, 60 implants placed in 54 patients were statistically analyzed in this randomized clinical trial (Fig. 1).

**Fig. 2:**
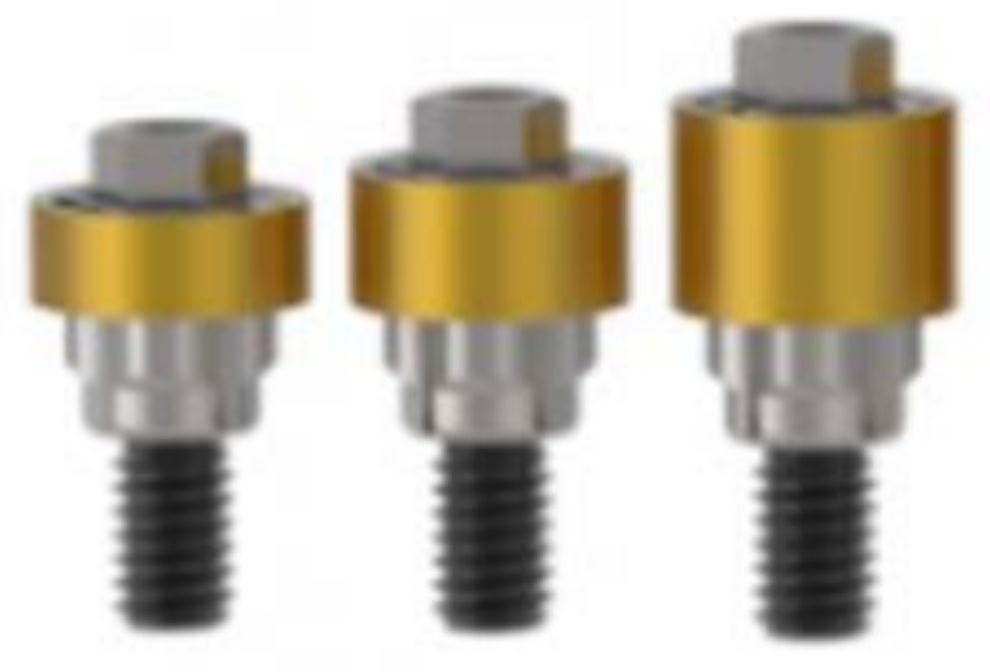
Anti-rotational intermediate abutments of 1.5, 2 and 3 mm for unitary restoration used in this trial

**Fig. 3:**
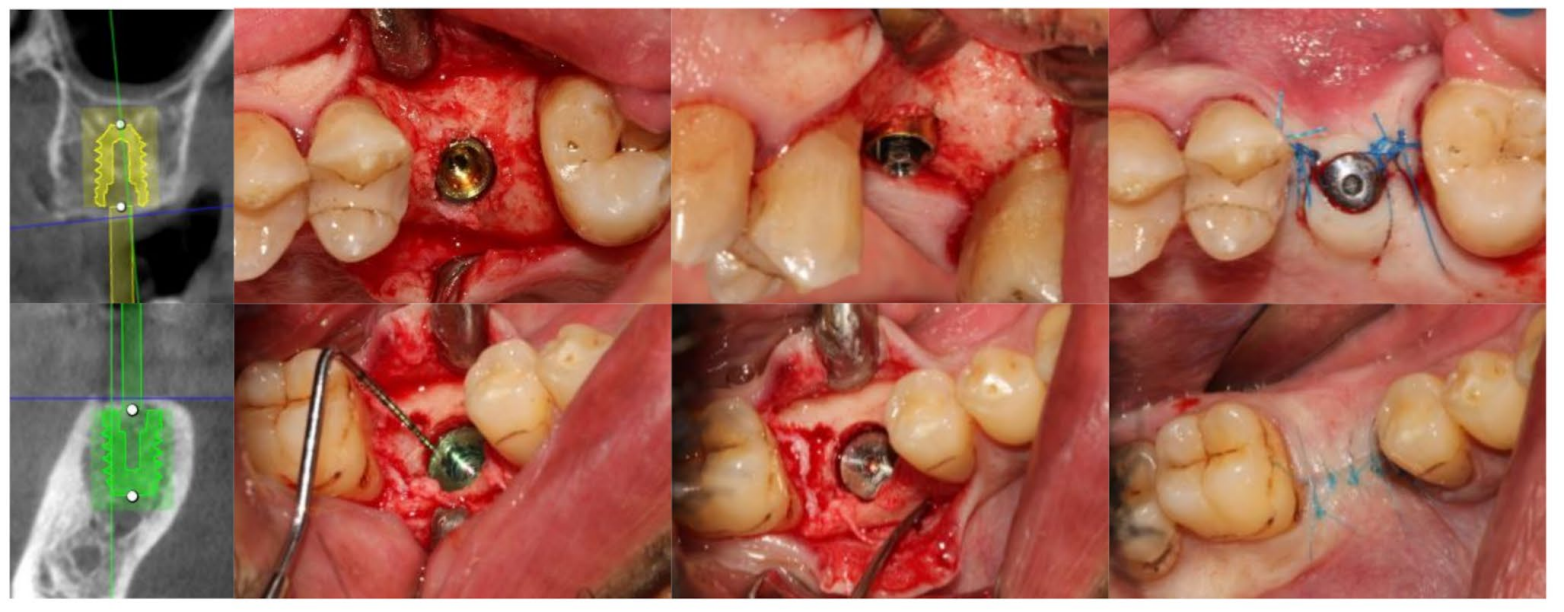
Selection of implant size through CBCT planning and clinical images of both protocols: “one abutment– one time” and “second surgery”

**Fig. 4:**
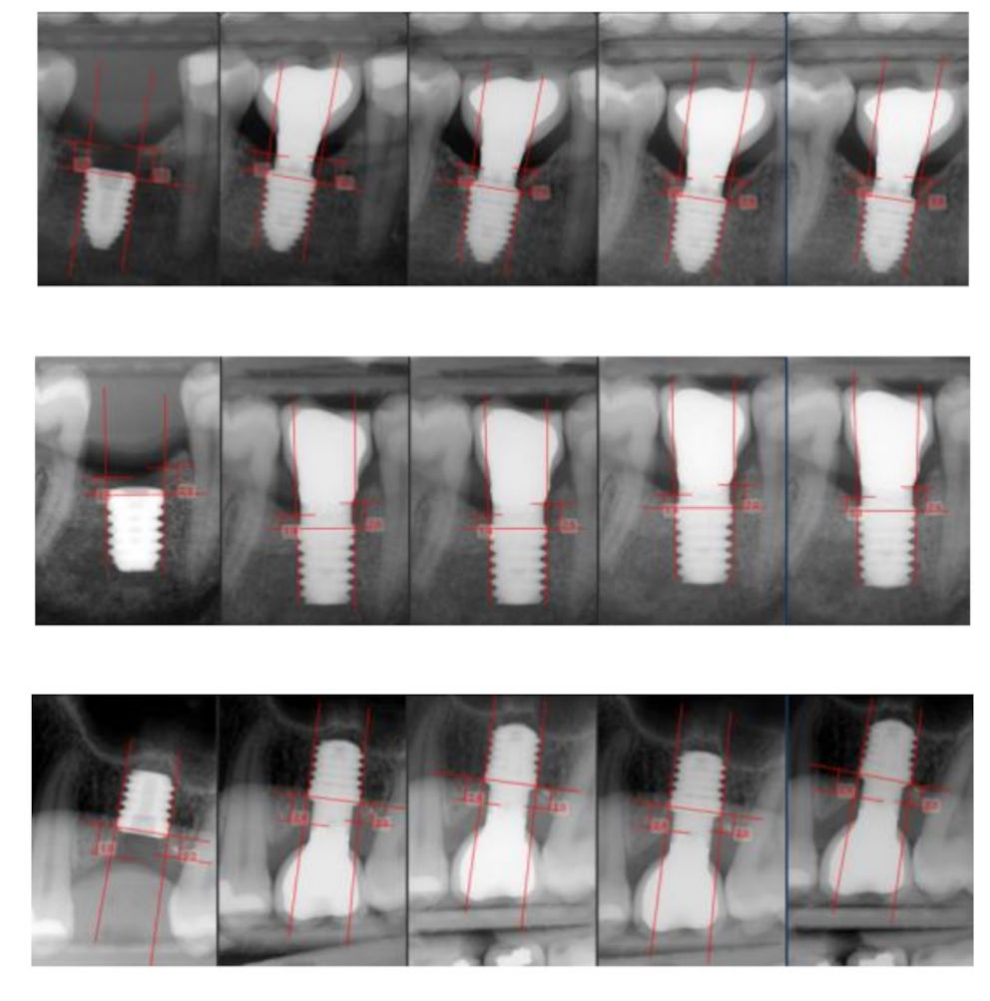
Radiographic images with follow-up for the 3 heights (1.5, 2 and 3 mm) at the 5 control points: implant surgery, crown placement, 3 months, 6 months and 12 months after loading

All installed implants were BTI UnicCa^®^ implants with a flat tetragonal internal connection (BTI Implant System, Vitoria, Spain). These implants had two connection diameters: 4.1 mm (narrow) and 5.5 mm (wide). The implant body diameter varied according to the available bone width and was classified as follows: for implants with a 4.1 mm connection, body diameters of 4.25, 4.5, 5.0, and 5.5 mm were used, whereas implants with a 5.5 mm connection had body diameters of 6.0 and 6.25 mm. All implants were restored with parallel anti-rotational intermediate abutments (Ti Golden^®^ UNIT, BTI Implant System, Vitoria, Spain), available in widths of 4.1 mm and 5.5 mm and heights of 1.5 mm, 2.0 mm, or 3.0 mm (Fig. 2). These abutments were either placed immediately during the installation surgery (one-phase protocol: “one abutment - one time”) or delayed until the second surgery, performed 8 weeks after implant installation (two-phase protocol: submerged implant) (Fig. 3). Standardized digital intraoral radiographs were taken for all patients using a silicone key and Rhin ring positioner, recorded at the time of implant installation (baseline), crown placement (T1), and at 3 (T2), 6 (T3), and 12 months after prosthetic loading (T4) (Fig. 4).

### Surgical procedures

Patients underwent a comprehensive clinical examination (including periodontal charting) and radiographic assessment (intraoral radiographs, orthopantomography, and CBCT). All surgical procedures were carried out by the same surgeon (JQH) under local anesthesia (Artinibsa^®^; Inibsa Dental SLU, Barcelona, Spain). The implant size was determined based on digital planning using CBCT (Planmeca Romexis Software), taking into account both bone quantity and quality. Prior to surgery, soft tissue thickness (from the mucosal surface to the crestal bone) at the implant placement site was measured using a periodontal probe (15 mm, PCP UNC 15; HuFriedy). A conventional placement protocol was followed, with delayed implant installation in a fully healed socket (minimum 16 weeks of healing following tooth extraction). Osteotomy was performed without irrigation at low speed (70 rpm), strictly adhering to the established protocol based on implant size and bone density. All implants were placed 1.5 mm subcrestally, following the established placement guidelines. Pharmacological prescriptions included antibiotics (Amoxicillin 1 g every 12 h for 7 days, or Clindamycin for those with allergies), anti-inflammatory medication (Dexketoprofen 25 mg every 8 h for 3 days), and probiotics (1 sachet per day for 1 week). Postoperative care instructions included applying pressure with gauze for 5 min, using ice for 10 min, a soft and cold diet for the first 24 h, and performing rinses with 0.12% chlorhexidine (Perioaid; Dentaid SL; Cerdanyola, Spain) twice daily for 7 days. Sutures were removed after 7 days post-surgery.

### Randomization

Implants were randomly allocated to one of the six treatment groups using a simple randomization method with a 1:1 allocation ratio, generated using SPSS version 23 (IBM Corp., Armonk, NY, USA). Random numbers were assigned to each implant site using the RV.UNIFORM function, and cases were then sorted and grouped accordingly into the six treatment arms. The random sequence was prepared by a member of the surgical team who was not involved in patient recruitment or any clinical procedures. Allocation was implemented through sealed, opaque envelopes that were opened immediately after implant placement by another independent member of the surgical team. This process ensured that both investigators and participants remained blinded to the allocation sequence. Based on the random assignment of abutment height and timing of placement, intermediate abutments of three different heights were screwed into the internal connection with a torque lower than the implant insertion torque. Abutments were placed either immediately during the same surgical procedure (one-phase protocol: “one-abutment-one-time”) or delayed, placed during the second surgery at 8 weeks post-insertion (two-stage protocol: submerged implant).

### Restorative procedures

Eight weeks after the implant installation surgery, the platforms of the submerged implants were exposed, and the intermediate abutment was connected with a torque of 35 Ncm. Ten weeks after surgery, the intermediate abutment torque was increased to 35 Ncm on the non-submerged implants, and the prosthetic phase commenced for all cases. All definitive implant-supported prostheses were single screw-retained metal-ceramic crowns. Laboratory records were obtained for all cases through digital intraoral scanning (iTero^®^; Align Technology Inc., San José, California, USA). The CAD design of the restorations was carried out using Exocad software. The CAM fabrication of the restorations involved milling the metal framework and layering the ceramic covering on a printed model. The single crowns were screwed in 2 weeks after the scanning phase with a prosthetic screw torque of 20 Ncm (conventional loading for all cases: 12 weeks after implant placement surgery). The screw access channels were sealed with Teflon and composite resin (GrandioSo; Voco GmbH; Cuxhaven, Germany). Occlusion was refined, and the adequacy of contact points and gingival sealing was verified. All restorative procedures were performed by the same prosthodontist (JQH).

### Radiographic evaluation

Standardized digital intraoral radiographs were obtained using a silicone key and Rhin ring positioner (Planmeca Prox + Planmeca Prosensor HD; Planmeca Oy; Helsinki, Finland) at implant placement (baseline), definitive crown placement (T1), and at 3 months (T2), 6 months (T3), and 12 months (T4) after functional loading. MBL measurements were performed using the Planmeca Romexis dental imaging software platform. Linear measurements were taken in each radiograph from the most mesial and distal points of the implant shoulder to the crestal bone. The magnification of the radiographs was corrected based on the radiographic measurement of the implant’s height and width, allowing each MBL measurement to be calibrated and recalculated accordingly. Radiograph acquisition, calibration, and measurement were all performed by a single calibrated examiner (JQH).

### Sociodemographic and clinical evaluation

The following sociodemographic data were recorded: age, gender (male/female), smoking habits (smoker/non-smoker), diabetes status (diabetic/non-diabetic), and history of periodontal disease (periodontal/non-periodontal). Implant-related data recorded included: implant body diameter (4.25, 4.5, 5.0, 5.5, 6.0, 6.25 mm), implant body length (6.5, 7.5, 8.5, 10.0, 11.5 mm), platform switching (PS; horizontal distance from the implant body surface to the prosthetic connection limit, defined as the difference between the body diameter and the connection diameter), implant location (maxilla/mandible), and quadrant (first or second premolar; first or second molar). Additionally, the following clinical data were recorded:

- Soft tissue thickness: measured from the mucosal surface to the crestal bone at the implant placement site using a periodontal probe (15 mm, PCP UNC 15; Hu-Friedy Mfg. Co. LLC; Frankfurt, Germany).
- Bone quality: classified according to Leckholm & Zarb (1985) as type I, II, III, or IV, and quantified by averaging the Hounsfield units at the implant placement site using Romexis Planmeca software.
- Implant survival (at T4).
- Complications (at T4). Mechanical complications: screw loosening, screw fracture, ceramic chipping or opening of the interproximal contact of the restoration; Biological complications: incision line dehiscence, peri-implant infection or postoperative pain; Aesthetic complications: exposure of the intermediate abutment, exposure of the restoration`s metal or inadequate ceramic color of the restoration.

### Periimplant health

To evaluate peri-implant health at the 12-month follow-up, two clinical parameters were recorded: probing depth (PD) and bleeding on probing (BOP). PD was measured in millimeters at six locations around each implant (three vestibular sites and three lingual/palatal sites) by first removing the screw-retained crown and using the prosthetic abutment platform as the reference point. BOP was assessed by observing any bleeding within 15 s post-probing. The presence or absence of BOP was calculated as the percentage of total probed sites (three vestibular sites and three lingual/palatal sites). The average of the six measurements was used for statistical analysis of both probing depth and bleeding.

### Overall patient satisfaction level

To assess overall patient satisfaction with the treatment outcome, each patient was surveyed at the end of the follow-up period (T4). The closed-ended question asked was: “Are you satisfied with the treatment outcome in terms of health, function, and aesthetics?” Responses were dichotomous: satisfied / not satisfied. The percentage of positive responses was calculated for statistical analysis.

### Statistical analysis

The qualitative covariates from the sociodemographic and clinical data were expressed as absolute and relative frequencies, while the quantitative covariates were presented as means and standard deviations. Significant prior differences were assessed using the Student’s t-test. Data distribution was confirmed to follow normality (*p* >.05) using the Shapiro-Wilk test (1965), allowing the application of parametric tests. A factorial repeated measures ANOVA was applied to assess marginal bone loss (MBL) over time, incorporating the covariates of platform switching, soft tissue thickness, implant diameter, and bone quality. Post-hoc analyses were conducted to compare the six treatment groups. The analysis included the F statistic from the ANOVA, degrees of freedom (df), p-value, effect size measured by partial eta squared (ηp²), and the confidence interval. The level of statistical significance was set at *p* <.05, with a 95% confidence interval. All analyses were performed using SPSS version 23 (SPSS; IBM; New York, USA).

## Results

### Sociodemographic and clinical data

The 54 patients included in this randomized clinical trial received a total of 60 implants, with an overall survival rate of 100% at 12 months. Table 1 summarizes the sociodemographic and clinical characteristics of the participants. The mean age of the patients was 50.35 ± 12.16 years, with 68.3% male and 31.7% female. Most patients were non-smokers (76.7%), none had diabetes, and 16.7% had a history of periodontal disease. Of the implants placed, 26 were located in the maxilla and 34 in the mandible. Regarding implant location, 6.7% were placed in first premolars, 21.7% in second premolars, 65% in first molars, and 6.7% in second molars. The most common implant diameter was 5.0 mm (33.3% of cases), and the most frequently selected implant length was 8.5 mm (48.3%). Most patients presented with type III bone quality (70%). Mechanical or biological complications were observed in 18.3% of the implants: prosthetic screw loosening (5 implants), ceramic chipping (2 implants), opening of the contact point (2 implants), incision line opening (1 implant), and postoperative pain (1 implant). No aesthetic complications were reported. The mean probing depth was 1.4 ± 0.45 mm, and the total percentage of bleeding on probing was 16.9 ± 22.5%. Additionally, 95% of the patients reported satisfaction with the treatment outcome. No statistically significant differences were found between the treatment groups across the analyzed variables, except for the soft tissue thickness variable.

**Table 1:** Clinical and sociodemographic variables

Variables	Group A3I (*n* = 10)	Group A2I (*n* = 10)	GroupA15I (*n* = 10)	Group A3D (*n* = 10)	Group A2D (*n* = 10)	GroupA15D (*n* = 10)	TOTAL	*P* value
**Age**	56.50 ± 12.60	51.30 ± 11.70	43.40 ± 12.33	51.10 ± 15.51	51.70 ± 08.41	48.10 ± 10.21	50.35 ± 12.16	**0.273**
**Gender**								**0.242**
**Male**	5 (50%)	9 (90%)	6 (60%)	8 (80%)	8 (80%)	5 (50%)	41 (68.3%)	
**Female**	5 (50%)	1 (10%)	4 (40%)	2 (20%)	2 (20%)	5 (50%)	19 (31.7%)	
**Smoking**								**0.560**
**Yes**	1 (10%)	1 (10%)	4 (40%)	3 (30%)	2 (20%)	3 (30%)	14 (23.3%)	
**No**	9 (90%)	9 (90%)	6 (60%)	7 (70%)	8 (80%)	7 (70%)	46 (76.7%)	
**Diabetes**								**-**
**Yes**	0 (0%)	0 (0%)	0 (0%)	0 (0%)	0 (0%)	0 (0%)	0 (0%)	
**No**	10 (100%)	10 (100%)	10 (100%)	10 (100%)	9 (90%)	10 (100%)	59 (98.3%)	
**Periodontal disease**								**0.598**
**Yes**	0 (0.0%)	2 (20%)	2 (20%)	1 (10%)	2 (20%)	3 (30%)	10 (16.7%)	
**No**	10 (100%)	8 (80%)	8 (80%)	9 (90%)	8 (80%)	7 (70%)	50 (83.3%)	
**Arch**								**0.678**
**Maxilla**	3 (30%)	7 (70%)	4 (40%)	3 (30%)	3 (30%)	6 (60%)	26 (43.3%)	
**Mandible**	7 (70%)	3 (30%)	6 (60%)	7 (70%)	7 (70%)	4 (40%)	34 (56.7%)	
**Position**								**0.073**
**First premolar**	0 (0.0%)	1 (10%)	1 (10%)	2 (20%)	0 (0.0%)	0 (0.0%)	4 (6.7%)	
**Second premolar**	1 (10%)	3 (30%)	3 (30%)	1 (10%)	0 (0.0%)	5 (50%)	13 (21.7%)	
**First molar**	6 (60%)	6 (60%)	6 (60%)	7 (70%)	10 (100%)	4 (40%)	39 (65%)	
**Second molar**	3 (30%)	0 (0.0%)	0 (0.0%)	0 (0.0%)	0 (0.0%)	1 (10%)	4 (6.7%)	
**Diameter**								**0.017**
**4.25 mm**	0 (0.0%)	2 (20%)	0 (0.0%)	3 (30%)	1 (10%)	0 (0.0%)	6 (10%)	
**4.5 mm**	1 (10%)	1 (10%)	6 (60%)	1 (10%)	0 (0.0%)	3 (30%)	12 (20%)	
**5.0 mm**	3 (30%)	3 (30%)	2 (20%)	4 (40%)	3 (30%)	5 (50%)	20 (33.3%)	
**5.5 mm**	3 (30%)	1 (10%)	2 (20%)	2 (20%)	1 (10%)	2 (20%)	11 (18.3%)	
**6.0 mm**	3 (30%)	2 (20%)	0 (0.0%)	0 (0.0%)	4 (40%)	0 (0.0%)	9 (15%)	
**6.25 mm**	0 (0.0%)	1 (10%)	0 (0.0%)	0 (0.0%)	1 (10%)	0 (0.0%)	2 (3.3%)	
**Length**								**0.056**
**6.5 mm**	2 (20%)	0 (0.0%)	0 (0.0%)	0 (0.0%)	1 (10%)	2 (20%)	5 (8.3%)	
**7.5 mm**	0 (0.0%)	1 (10%)	1 (10%)	1 (10%)	2 (20%)	1 (10%)	6 (10%)	
**8.5 mm**	6 (60%)	2 (20%)	9 (90%)	3 (30%)	4 (40%)	5 (50%)	29 (48.3%)	
**10 mm**	1 (10%)	5 (50%)	0 (0.0%)	5 (50%)	3 (30%)	1 (10%)	15 (25%)	
**11.5 mm**	1 (10%)	2 (20%)	0 (0.0%)	1 (10%)	0 (0.0%)	1 (10%)	5 (8.3%)	
**Platform Switching**	0.88 ± 0.40	0.65 ± 0.39	0.70 ± 0.42	0.72 ± 0.49	0.70 ± 0.34	0.85 ± 0.37	0.75 ± 0.39	**0.771**
**Soft tissue thickness (mm)**	3 ± 0.33	2.6 ± 0.65	3.35 ± 0.88	2.9 ± 0.99	2.2 ± 0.67	2.9 ± 0.61	2.82 ± 0.78	**0.022**
**Bone quality**								**0.664**
**1**	0 (0.0%)	2 (20%)	0 (0.0%)	1 (10%)	0 (0.0%)	0 (0.0%)	3 (5%)	
**2**	3 (30%)	0 (0.0%)	0 (0.0%)	1 (10%)	2 (20%)	2 (20%)	8 (13.3%)	
**3**	7 (70%)	5 (50%)	8 (80%)	7 (70%)	8 (80%)	7 (70%)	42 (70%)	
**4**	0 (0.0%)	3 (30%)	2 (20%)	1 (10%)	0 (0.0%)	1 (10%)	7 (11.7%)	
**Complications**	2 (20%)	2 (20%)	0 (0.0%)	2 (20%)	3 (30%)	2 (20%)	11 (18.3%)	**0.689**
**Mechanical**	2 (20%)	2 (20%)	0 (0.0%)	1 (10%)	2 (20%)	2 (20%)	9 (15%)	
**Biological**	0 (0.0%)	0 (0.0%)	0 (0.0%)	1 (10%)	1 (10%)	0 (0.0%)	2 (3.3%)	
**Aesthetic**	0 (0.0%)	0 (0.0%)	0 (0.0%)	0 (0.0%)	0 (0.0%)	0 (0.0%)	0 (0.0%)	
**PD (mm)**	1.15 ± 0.41	1.25 ± 0.49	1.5 ± 0.53	1.4 ± 0.39	1.45 ± 0.44	1.65 ± 0.41	1.4 ± 0.45	**0.179**
**BOP (%)**	8.5 ± 14.4	11.9 ± 14	11.9 ± 14	25.2 ± 33.6	15.2 ± 21.6	26.9 ± 29.6	16.9 ± 22.5	**0.351**
**Satisfaction**	10 (100%)	10 (100%)	9 (90%)	9 (90%)	10 (100%)	9 (90%)	57 (95%)	0.502

### Radiographic evaluation of MBL

Table 2 presents the radiographic data for total MBL (mean MBL between mesial and distal measurements) by group 12 months after prosthetic restoration placement. The lowest MBL values were observed in Groups **A3I** (0.13 ± 0.11 mm) and **A2I** (0.24 ± 0.11 mm), with no statistically significant differences between these two groups (*p* >.05). Groups **A15I** (0.70 ± 0.12 mm), **A3D** (0.66 ± 0.11 mm), **A2D** (0.62 ± 0.12 mm), and **A15D** (0.78 ± 0.11 mm) exhibited significantly higher MBL compared to Groups **A3I** and **A2I** (*p* <.05), with no statistically significant differences among these four groups (*p* >.05). Table 3 presents the differences in total mean MBL among the six groups.

**Table 2:** Cumulative mean MBL by group after 12 months of functional loading. Note: the covariables included in the model were evaluated at the following values: PS = 0.7517, gingiva = 2.8250, diameter = 5.1150, bone = 2.8833

Group	Mean	Standard Error	95% Confidence Interval	
			Lower Limit	Upper Limit
**Group A3I**	0.127	0.111	-0.095	0,349
**Group A2I**	0.235	0.110	0.015	0,455
**Group A15I**	0.700	0.119	0.461	0,939
**Group A3D**	0.664	0.110	0.442	0,886
**Group A2D**	0.618	0.124	0.369	0,867
**Group A15D**	0.781	0.110	0.560	1,002

**Table 3:** Mean differences in MBL among the six groups, based on estimated marginal means. Note: the covariables included in the model were evaluated at the following values: PS = 0.7517, gingiva = 2.8250, diameter = 5.1150, bone = 2.8833

(I) Group	(J) Group	Mean Difference (I-J)	Std. Error	Sig. b	95% CI: Lower Limit	95% CI: Upper Limit
Group A3I	Group A2I	-0.108	0.156	0.491	-0.421	0.205
	Group A15I	-0.573*	0.166	0.001	-0.907	-0.239
	Group A3D	-0.537*	0.159	0.001	-0.856	-0.218
	Group A2D	-0.490*	0.163	0.004	-0.819	-0.162
	Group A15D	-0.654*	0.158	0.000	-0.970	-0.337
Group A2I	Group A3I	0.108	0.156	0.491	-0.205	0.421
	Group A15I	-0.465*	0.167	0.008	-0.801	-0.129
	Group A3D	-0.429*	0.158	0.009	-0.746	-0.111
	Group A2D	-0.382*	0.157	0.019	-0.698	-0.067
	Group A15D	-0.546*	0.159	0.001	-0.864	-0.227
Group A15I	Group A3I	0.573*	0.166	0.001	0.239	0.907
	Group A2I	0.465*	0.167	0.008	0.129	0.801
	Group A3D	0.036	0.157	0.817	-0.278	0.351
	Group A2D	0.083	0.189	0.663	-0.296	0.462
	Group A15D	-0.081	0.157	0.609	-0.395	0.234
Group A3D	Group A3I	0.537*	0.159	0.001	0.218	0.856
	Group A2I	0.429*	0.158	0.009	0.111	0.746
	Group A15I	-0.036	0.157	0.817	-0.351	0.278
	Group A2D	0.046	0.174	0.792	-0.304	0.396
	Group A15D	-0.117	0.152	0.445	-0.423	0.188
Group A2D	Group A3I	0.490*	0.163	0.004	0.162	0.819
	Group A2I	0.382*	0.157	0.019	0.067	0.698
	Group A15I	-0.083	0.189	0.663	-0.462	0.296
	Group A3D	-0.046	0.174	0.792	-0.396	0.304
	Group A15D	-0.163	0.174	0.352	-0.512	0.186
Group A15D	Group A3I	0.654*	0.158	0.000	0.337	0.970
	Group A2I	0.546*	0.159	0.001	0.227	0.864
	Group A15I	0.081	0.157	0.609	-0.234	0.395
	Group A3D	0.117	0.152	0.445	-0.188	0.423
	Group A2D	0.163	0.174	0.352	-0.186	0.512

**Fig. 5:**
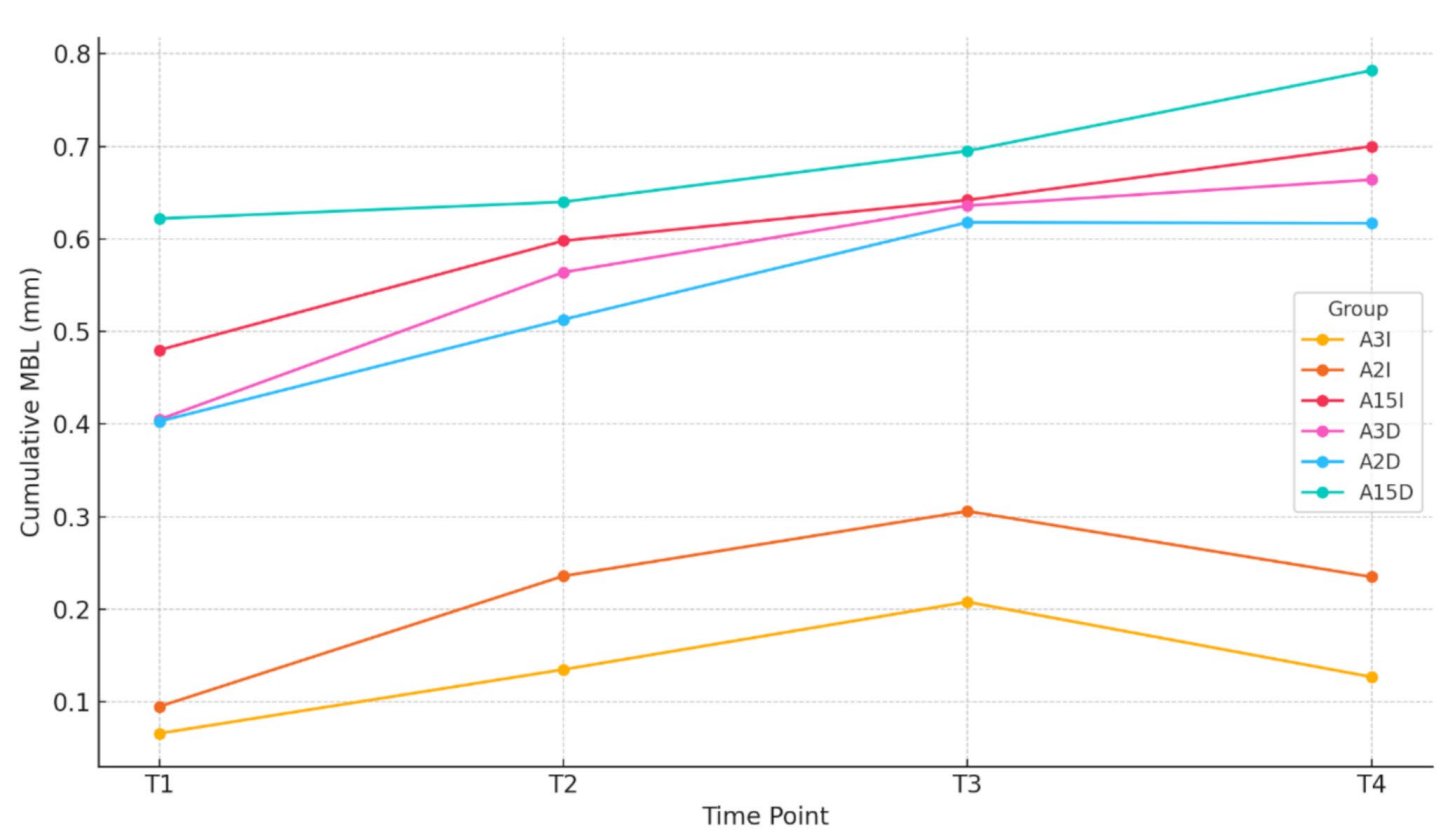
Cumulative MBL for each group over time. Lines represent the progression of mean cumulative MBL across four time points (T1–T4) for each treatment group. Note: The covariables included in the model were evaluated at the following values: PS = 0.7517, gingiva = 2.8250, diameter = 5.1150, bone = 2.8833

**Fig. 6:**
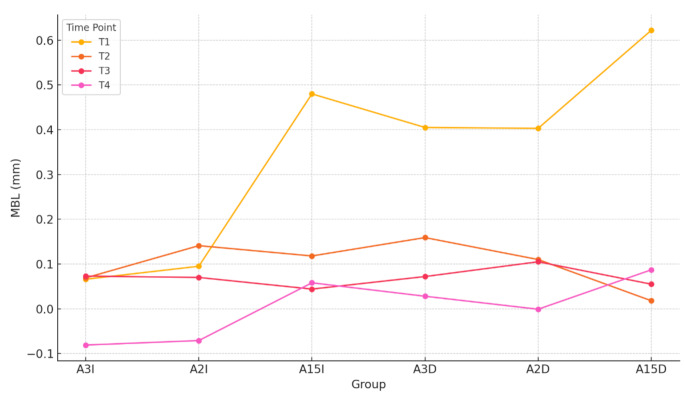
Non-cumulative MBL at each time point by group. Lines represent the mean MBL at each time point (T1–T4) for each treatment group, allowing comparison of temporal changes in bone loss across groups. Note: The covariables included in the model were evaluated at the following values: PS = 0.7517, gingiva = 2.8250, diameter = 5.1150, bone = 2.8833

Table 4 displays the radiographic data for partial MBL (mean MBL between mesial and distal measurements) by group across the four measurement time points. At T1, the lowest MBL values were observed in Groups **A3I** and **A2I**, with no statistically significant differences between these two groups; Groups **A15I**, **A3D**, **A2D**, and **A15D** exhibited significantly higher MBL compared to Groups **A3I** and **A2I** (*p* <.05), with no statistically significant differences among them (*p* >.05). At T2 and T3, no statistically significant differences in MBL were observed among the six groups. At T4, Groups **A3I** and **A2I** demonstrated significantly lower MBL compared to Group **A15D** (*p* <.05), and Group **A3I** also showed lower MBL compared to Group **A15I** (*p* <.05). Figure 5 illustrates the variation in cumulative MBL for each group over time. Figure 6 shows the non-cumulative MBL at each time point as a function of each group.

**Table 4:** Partial mean MBL by group for each of the 4 measurement times. Note: the covariables included in the model were evaluated at the following values: PS = 0.7517, gingiva = 2.8250, diameter = 5.1150, bone = 2.8833

Group	Mean	Std. Error	95% Confidence Interval		Group	Mean	Std. Error	95% Confidence Interval	
			Lower Bound	Upper Limit				Lower Bound	Upper Limit
T1					T3				
**Group A3I**	0.066	0.083	-0.100	,232	**Group A3I**	0.073	0.041	-0.010	0.156
**Group A2I**	0.095	0.082	-0.069	,260	**Group A2I**	0.070	0.041	-0.012	0.152
**Group A15I**	0.480	0.089	0.301	,659	**Group A15I**	0.044	0.044	-0.045	0.133
**Group A3D**	0.405	0.083	0.239	,570	**Group A3D**	0.072	0.041	-0.010	0.155
**Group A2D**	0.403	0.093	0.216	,589	**Group A2D**	0.105	0.046	0.013	0.198
**Group A15D**	0.622	0.082	0.457	,787	**Group A15D**	0.055	0.041	-0.027	0.137
**T2**					**T4**				
**Group A3I**	0.069	0.063	-0.058	,196	**Group A3I**	-0.081	0.044	-0.170	0.008
**Group A2I**	0.141	0.062	0.015	,266	**Group A2I**	-0.071	0.044	-0.159	0.017
**Group A15I**	0.118	0.068	-0.018	,255	**Group A15I**	0.058	0.048	-0.038	0.154
**Group A3D**	0.159	0.063	0.033	,286	**Group A3D**	0.028	0.044	-0.061	0.117
**Group A2D**	0.110	0.071	-0.032	,252	**Group A2D**	-0.001	0.050	-0.100	0.099
**Group A15D**	0.018	0.063	-0.109	,144	**Group A15D**	0.087	0.044	-0.002	0.175

## Discussion

The primary objective of this randomized clinical trial was to evaluate the influence of intermediate abutment height and placement timing on MBL after one year of loading on single screw-retained crowns on platform-switched implants placed 1.5 mm subcrestally. The results demonstrated significantly greater MBL when intermediate abutments of 1.5 mm height were placed at the time of implant installation (first surgery) compared to intermediate abutments of 2 mm and 3 mm height. Furthermore, when intermediate abutments were placed delayed (second surgery), MBL was significantly greater, regardless of height (1.5 mm, 2 mm, or 3 mm), compared to the immediate placement of 2 mm and 3 mm abutments (Fig. 5).

These findings suggest that the “one abutment– one time” protocol may **contribute to the preservation of** peri-implant marginal bone levels. The results are consistent with other studies comparing this protocol to the standard prosthetic protocol, showing a reduction in MBL of approximately 0.5 mm at 12 months [26–29]. Bressan et al. [30] demonstrated a similar difference in a 36-month study, indicating that the difference is more pronounced during the initial months of healing, as shown by the findings of this trial. Upon comparing both protocols over a 5-year period, Sanz et al. did not observe statistically significant differences in the radiographic interproximal bone levels, concluding that the connection and disconnection of healing abutments are not associated with an increased long-term MBL [31]. Similarly, increasing the height of the intermediate abutment placed at the time of surgery reduces MBL, a result consistent with the literature indicating that shorter abutments (< 2 mm) lead to greater MBL compared to longer abutments (≥ 2 mm). Blanco et al. [32] and Pico et al. [33] reported greater MBL with 1 mm abutments compared to 3 mm abutments in follow-ups of 6 and 12 months, respectively. The first study also showed an association between smoking and MBL. Spinato et al. [34] and Muñoz et al. [35] reported similar results for the same abutment heights, with the first study ruling out the association between mucosal thickness and MBL, and the second concluding that this association is minimized by the subcrestal position. The meta-analysis conducted by the same author reinforces this conclusion, demonstrating a reduction in marginal bone loss (MBL) in two-piece implants with long abutments (≥ 2 mm), with the impact of abutment height being attenuated when the implant is placed at a subcrestal level [36]. The vertical shift towards the coronal aspect of the abutment-restoration interface, which distances the gap from the peri-implant bone, may explain the positive effect of greater abutment height.

In this trial, two implant-related covariates were considered when evaluating MBL: platform switching and diameter. Platform switching implants contribute to the preservation of marginal bone levels, which may be associated with the establishment of a favorable horizontal peri-implant biological width [4]. The extent of platform switching does not appear to have a significant influence on marginal bone levels according to the results, but it does enable predictable placement of the implant at a subcrestal level in all cases, horizontally distancing the implant-abutment interface from the bone crest. Implant diameter also did not have a statistically significant impact on MBL. In this context, Canullo et al. [37] concluded that bone resorption is more closely related to biological factors (restoration of biological width) than to biomechanical factors (distribution of forces to the bone).

Two additional host-related covariates were considered when analyzing MBL: soft tissue thickness and bone quality. Regarding soft tissue thickness, its influence on MBL was not statistically significant. According to Linckevicius et al., [38] platform switching does not prevent MBL if mucosal tissue thickness is less than 2 mm at the time of implant placement. Therefore, placing the implant 1.5 mm subcrestally may help prevent subsequent remodeling from exposing the implant body, which always occurs coronally to the platform. Bone quality also did not significantly influence MBL. Insua et al. [11] associate a lower proportion of spongy bone with greater MBL, which may be explained by high insertion torque in the presence of a thick cortical layer. A customized drilling protocol, considering implant size and bone quality of the receiving site, allows for atraumatic osteotomies that minimize early remodeling.

Regarding the evolution of marginal bone loss (MBL) over time, this study demonstrates that the majority of bone loss occurs within the first few months, between implant placement surgery (baseline) and crown placement (T1) (Fig. 6). Galindo et al. [2] reached similar conclusions, stating that the greatest MBL occurs at early stages, during the interval between abutment connection and crown placement. The same author [39] defines a threshold of 0.5 mm of accumulated MBL within the 6-month follow-up period after loading as an indicator of progression. The 10-year study by Windael et al. also identifies an early bone loss threshold of 0.5 mm as a significant predictor of peri-implant pathology, with implants exceeding this limit exhibiting a 5.43-fold higher probability of developing peri-implantitis, especially when additional risk factors such as smoking or a history of periodontal disease are present [40]. For all groups except Groups 1 and 2, MBL at 6 months exceeds the 0.5 mm threshold. Therefore, the combination of short abutments with immediate placement or the combination of abutments of any height with delayed placement can be excluded as valid clinical alternatives. However, the immediate placement of long abutments (2–3 mm) significantly reduces MBL during the critical phase between implant placement surgery and crown placement, maintaining values below the 0.5 mm threshold throughout the follow-up period. The combination of long abutments with immediate placement represents the optimal clinical approach.

There are several limitations associated with this research. Although standardized, radiographic evaluation is two-dimensional, thus only assessing MBL in the mesiodistal plane. Furthermore, although the results did not show a significant influence, both the diameter and extent of implant platform changes are highly variable. While the statistical analysis ruled out any significant effect, a notable difference in means was observed when analyzing the soft tissue thickness variable. The follow-up period was 12 months, which, according to the literature and as this study also concludes, encompasses the majority of MBL. The absence of a priori sample size calculation constitutes a methodological limitation to consider when interpreting the results and conclusions of this study. Therefore, future studies should incorporate randomized long-term follow-ups with larger sample sizes to confirm or refute the benefits of using immediately placed intermediate abutments of 2 mm or greater for single-tooth replacement at the time of implant installation surgery.

## Conclusions

Immediate placement of a 3 mm single intermediate abutment at the time of surgery results in statistically significantly less peri-implant marginal bone loss compared to the immediate placement of a 1.5 mm abutment after one year of follow-up, with no significant difference when compared to the immediate placement of a 2 mm abutment. Additionally, the immediate placement of a single intermediate abutment of 2–3 mm height shows lower rates of peri-implant marginal bone loss compared to the delayed placement of abutments of any height (1.5 mm, 2 mm, or 3 mm).

## Data Availability

No datasets were generated or analysed during the current study.
